# Transcriptional control of ILC identity

**DOI:** 10.3389/fimmu.2023.1146077

**Published:** 2023-03-09

**Authors:** Anna A. Korchagina, Sergey A. Shein, Ekaterina Koroleva, Alexei V. Tumanov

**Affiliations:** Department of Microbiology, Immunology and Molecular Genetics, University of Texas Health Science Center at San Antonio, San Antonio, TX, United States

**Keywords:** innate lymphoid cells, transcriptional regulation, ILC identity, ILC plasticity, transcription factor

## Abstract

Innate lymphoid cells (ILCs) are heterogeneous innate immune cells which participate in host defense, mucosal repair and immunopathology by producing effector cytokines similarly to their adaptive immune cell counterparts. The development of ILC1, 2, and 3 subsets is controlled by core transcription factors: T-bet, GATA3, and RORγt, respectively. ILCs can undergo plasticity and transdifferentiate to other ILC subsets in response to invading pathogens and changes in local tissue environment. Accumulating evidence suggests that the plasticity and the maintenance of ILC identity is controlled by a balance between these and additional transcription factors such as STATs, Batf, Ikaros, Runx3, c-Maf, Bcl11b, and Zbtb46, activated in response to lineage-guiding cytokines. However, how interplay between these transcription factors leads to ILC plasticity and the maintenance of ILC identity remains hypothetical. In this review, we discuss recent advances in understanding transcriptional regulation of ILCs in homeostatic and inflammatory conditions.

## 1 Introduction

**Figure 1:**
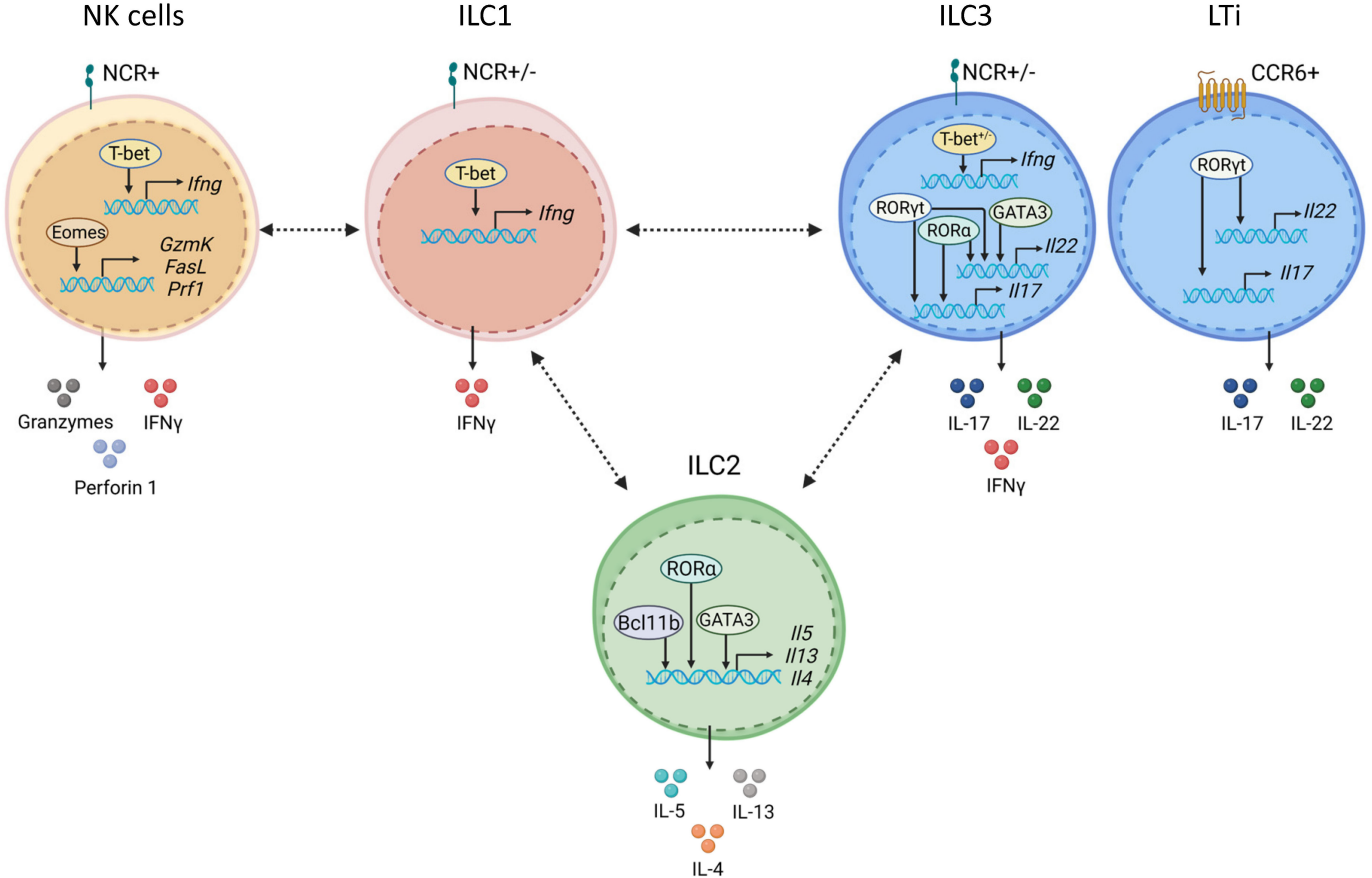
ILC subsets are defined by core transcription factors. NK cells express natural cytotoxicity receptors (NCRs) and require Eomes and T-bet for their development. Eomes and T-bet maintain NK cell effector functions by inducing granzymes, perforin 1, and IFNγ. ILC1 development and maintenance depends on T-bet, which is required for IFNγ production. ILC2s require GATA3 for their development and maintenance. Bcl11b promotes ILC2 development by controlling ILC2 lineage associated genes and by restraining ILC3 development. RORα, Bcl11b and GATA3 control production of type 2 effector cytokines, IL-5, IL-13 and IL-4 in ILC2. ILC3s and LTi cells express RORγt, which is required for their development and maintenance. RORγt with RORα induce type 3 effector cytokines, IL-17 and IL-22. ILC3s are divided on NCR^+^ and NCR^-^ subsets. Development of NCR^+^ ILC3s depends on GATA3 and T-bet. GATA3 together with RORγt regulate IL-22. LTi cells express CCR6. Plasticity between ILC subsets is shown by dashed black arrows.

Innate lymphoid cells (ILCs) are enriched in mucosal tissues where they control tissue homeostasis and rapidly respond to invading pathogens (1). Upon activation ILCs produce effector cytokines to orchestrate host defense at the early stage of infection. ILCs are currently classified into five subsets based on their development and effector functions: ILC1, ILC2, ILC3, lymphoid tissue inducer (LTi) cells and natural killer (NK) cells (2). Classification of ILCs is based on the network of lineage-determining transcription factors (LDTFs) which activate or repress genes defining the cellular identity of ILCs (3–10). Moreover, LDTFs regulate subset-specific genes which define migratory and metabolic cell features as well as effector functions of ILCs (5, 11, 12). Numerous transcription factors (TFs) control the development of ILCs from common lymphoid progenitors in the bone marrow (6). Activation or repression of the specific gene expression depends on the synergistic effects of numerous TFs which can bind to specific DNA sequences within the gene promoter area (13). The accessibility of designated DNA motif to TFs is controlled by the chromatin structure. Recently it has been shown that the development and differentiation of ILC subsets depend on the three-dimensional genome organization that regulates chromatin accessibility and gene expression in ILCs (14). ILC subsets identity depends on the activation or repression of the key transcription factors T-bet, GATA3 and RORγt (**Figure 1**). Accumulating evidence suggests that, in addition to these core factors, additional TFs define ILC identity and plasticity. Therefore, in this review, we will discuss recent advances in understanding the transcriptional regulation of ILC identity and plasticity in homeostatic and inflammatory conditions and provide visual presentation of potential mechanisms.

## 2 Transcriptional control of ILC development

NK cells and ILC1s are closely related ILC subsets which are characterized by expression of T-bet (encoded by *Tbx21* gene) and production of IFNγ/TNF upon activation (15–17). NK cells and ILC1s contribute to early host defense against intracellular pathogens and viruses (15). For example, during *Toxoplasma gondii* infection, NK cells and ILC1s contribute to protection by producing IFNγ, while ILC1s also express high levels of TNF (15). Interestingly, IFNγ and TNF-producing ILC1s are increased in the intestinal tissue of Crohn’s disease patients (18, 19), where TNF together with IFNγ can increase permeability of the intestinal epithelial barrier leading to exacerbation of inflammation (20–22). NK cells are migratory cytotoxic cells, whereas ILC1s are mainly considered as tissue-resident cells (15, 23, 24). IL-15 is required for the development and maintenance of both NK cells and ILC1s (15, 25). NK cells can exist in the tissues in different maturation states with distinct cytotoxic capacity and ability to produce cytokines. The maturation states are defined according to the group of markers that they express, such as acquiring integrin αM (CD11b) and downregulating CD27 markers (26, 27). Immature NK cells (CD27^+^ CD11b^-^) develop to terminally differentiated NK cells (CD27^-^ CD11b^+^ KLRG1^+^) with high cytotoxic capacity and with the ability to produce IFNγ (28–30). Transcription factors Eomes and T-bet are critical for normal development and maturation of NK cells (15, 31, 32). Eomes is expressed during all stages of NK cell development, whereas T-bet expression is increased only in mature NK cells (33–35). The ratio between Eomes and T-bet is critical for proper NK cell differentiation, maturation and function (31). Thus, deletion of both Eomes and T-bet leads to the complete loss of NK cells while single TF deficiency causes an immature NK cell phenotype suggesting that both TFs are essential for NK cell development (34, 35). Moreover, inducible deletion of Eomes in mature NK cells leads to their loss whereas ILC1s are preserved, suggesting that Eomes is necessary not only for the development but also for the maintenance of NK cells (36). A recent study demonstrated that during early NK cell differentiation Eomes induces expression of the genes responsible for NK cell survival, such as IL-2Rβ and IL-15Rβ (31). Conversely, T-bet controls terminal stages of NK cell differentiation (31). The analysis of Eomes/T-bet responsive genes showed that Eomes induces the NK cell-specific gene expression whereas T-bet regulates expression of the broader gene array (31). For example, Eomes induced expression of the genes responsible for NK cell cytotoxicity (*FasL, GzmK, Klra8, Prf1*) whereas T-bet induced expression of the genes which regulate responsiveness to IL-12 (*Il18r1, Il12*rβ*2, Ifnγ*) (31, 36). Both T-bet and Eomes are positive regulators of NK cell maturation while Bach2 TF is a negative regulator of terminal differentiation of NK cells (29, 30). The expression of Bach2 decreases in immature compared to terminally differentiated NK cells (29, 30). Moreover, Bach2 deficiency led to upregulation of genes responsible for NK cell effector functions such as granzyme B and KLRG1, suggesting that Bach2 can control NK cell maturation through repression of effector genes (30).

In contrast to NK cells, differentiation of ILC1s depends on T-bet, whereas Eomes is dispensable for ILC1 development (15, 33, 37). Although ILC1s do not express Eomes, a recent study described ILC1 population with cytotoxic activity in the liver and salivary gland, which had low levels of Eomes expression (38). These cells produced granzyme C and were distinct from NK cells (38). Interestingly, the majority of ILC1s in the liver and spleen express or have a history of granzyme C expression (38). Granzyme C production by ILC1s is dependent on T-bet, but not Eomes (38).

ILC2s participate in immune response against helminths by producing type two cytokines IL-4, IL-13, IL-5 and IL-9 (39–42). Additionally, ILC2s are implicated in the pathogenesis of respiratory and skin diseases (43–45). ILC2s promote restoration of damaged mucosal tissue by producing amphiregulin (AREG) (46–48). Although all ILCs express low levels of GATA3, ILC2s display the highest levels of GATA3 expression (10, 11, 15, 49), which is required for their development and maintenance (50–53). Similar to Th2 cell development, GATA3 can promote ILC2 lineage fate determination by suppressing RORγt expression in these cells (10, 54). Moreover, ILC2 differentiation depends on Notch signaling and availability of IL-7 (55). ILC2 precursors have been identified in bone marrow and thymus (56, 57). Activation of Notch signaling induces differentiation of thymic progenitor cells to ILC2s (58).

Another TF participating in ILC2 development is RORα (55, 56). It was demonstrated that in the thymus RORα is critical for ILC2 development by suppressing T cell commitment (56). Thus, overexpression of RORα induced ILC2 development while the absence of RORα promoted differentiation of the precursor cells to T cell lineage (56). In addition, it was shown that *Rora* binds to promoters of *Il13* and *Il5* genes in ILC2s, suggesting that RORα can promote effector function of ILC2s (56). Moreover, high RORα expression was demonstrated in all ILC subsets using five-color polychromic ILC reporter mice, suggesting the role of RORα in the regulation of other ILC subsets (59).

Bcl11b is another transcription factor that promotes ILC2 development by stabilizing expression of ILC2 lineage-associated genes and restricting their differentiation into ILC3s (60, 61). It was shown that Bcl11b binds to different sites of *Il4*, *Il13* and *Il5* genes in ILC2s (62). Additionally, Bcl11b deficient ILC2s display a decreased expression of IL-4, IL-13 and IL-5 (62). Furthermore, ILC2s were reduced in peripheral blood of patients with heterozygous germline Bcl11b mutation supporting the essential role of Bcl11b in ILC2 development (63). Interestingly, although Bcl11b is expressed in both T cells and ILC2s during development, it regulates distinct set of genes in these cells (62). However, how Bcl11b cooperate with GATA3 in the regulation of ILC2 specific genes remains to be further determined.

ILC3 development depends on RORγt (encoded by *Rorc* gene) (64). ILC3s contribute to the host defense against extracellular pathogens, fungi, and maintenance of epithelial cell homeostasis by producing IL-22 and IL-17 (65). Moreover, ILC3s can protect intestinal epithelial cells from TNF-induced apoptosis by producing heparin-binding EGF-like growth factor (66). ILC3s can be subdivided into two distinct subpopulations of CCR6^+^ and CCR6^-^ cells (67). CCR6^+^ ILCs include LTi (fetal lymphoid tissue inducer) cells and LTi-like cells (67). Although both ILC3s and LTi cells depend on RORγt expression for their development, LTis are considered as a separate lineage (64, 68). ILC3s develop from the progenitors that express TF PLZF, whereas LTi cells originate from the progenitors with no history of PLZF expression (9, 68). LTi cells control lymphoid organogenesis during embryonic development by producing lymphotoxin (LT) and TNF, whereas phenotypically close LTi-like cells appear in adulthood (64, 69–72). CCR6^-^ ILC3s are derived from another precursor and their development depends not only on RORγt but also on TF Ahr (67, 68). Some CCR6^-^ ILC3s express NKp46 (the member of natural cytotoxicity receptors, NCRs) and depend on T-bet, which is required for their development (67, 73, 74). GATA3 is required for the development of CCR6^-^ ILC3s but not LTi cells (10). It was proposed that during ILC ontogeny, GATA3 expression determines the divergence of LTis and other ILC subsets (10). In addition to controlling ILC3 commitment, GATA3 maintains mature ILC3 homeostasis (10). GATA3 controls the expression of IL-7Rα in ILC3s to promote cell survival and proliferation, similarly to its role in ILC2s and Th2 cells (10, 52, 75).

## 3 Transcriptional control of ILC plasticity

Extensive research over the past decade revealed plasticity within all ILC subsets that is largely controlled by tissue-derived factors. Thus, inflammation, infection or changing environmental conditions lead to activation of numerous intracellular signaling pathways that induce production of cytokines which in turn induce changes in ILC phenotypes and their function (18, 76). Therefore, ILC plasticity allows tissue resident cells to quickly adjust to the changes upon pathogen invasion or inflammatory conditions that could require different types of immune responses at the different stages of disease. It is becoming evident that ILC plasticity is not only an important driver of protective immune responses but can also lead to exacerbation of chronic and inflammatory diseases (19, 43, 45, 77). Accumulating evidence suggests that ILC plasticity can be reversed, underlying the existence of the mechanisms maintaining ILC balance under physiological conditions and during pathogen invasion to prevent excessive inflammation (76, 78). However, the transcriptional drivers of ILC plasticity remain poorly understood.

During pathogen invasion or ongoing inflammation cytokine production by immune and non-immune cells drives ILC plasticity by regulating the expression of lineage-determining transcription factors which, in turn induce production of effector cytokines by ILCs (18, 19, 76, 79). The ability of ILCs to undergo plasticity in response to distinct cytokines depends on the surface expression of appropriate cytokine receptors (80). Activated epithelial cells or myeloid cells produce type 1 cytokines such as IL-18, IL-15 and IL-12 which stimulate IFNγ production by ILC1s, whereas IL-23, IL-1β and IL-2 trigger IL-22 and IL-17 secretion by ILC3s (18, 79, 81). Distinct cytokine combinations induce phenotypical changes in the ILCs, leading to their functional plasticity.

**Figure 2:**
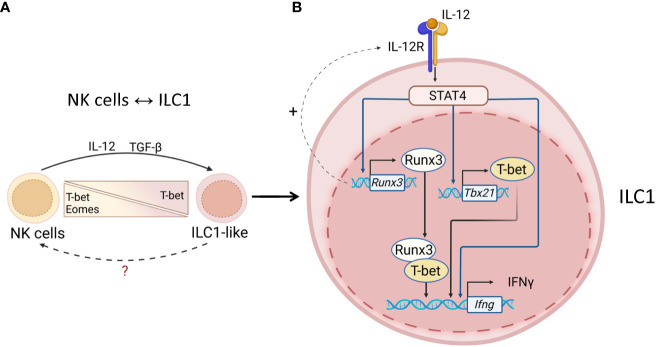
Transcriptional regulation of ILC1s and NK cells. **(A)** NK↔ILC1 plasticity. Tumors and mucosal pathogens, such as toxoplasma can induce IL-12 and TGF-β production by DCs and macrophages to drive NK↔ILC1 plasticity. NK↔ILC1 conversion is controlled by upregulation of T-bet and downregulation of Eomes. The ability of ILC1-like cells to convert back to NK cells remains unknown. **(B)** Maintenance of ILC1 identity. T-bet is required for ILC1s to produce IFNγ. IL-12R signaling activates STAT4, which induces T-bet transcription. Activated STAT4 also binds to Runx3 and IFNγ promoters to induce expression. Runx3 can also promote expression of IL12Rb1 thereby amplifying IL-12R signaling. T-bet associates with Runx3 to drive IFNγ transcription. IL-12R/STAT4 signaling maintains ILC1 phenotype *via* inducing T-bet and Runx3 for IFNγ production.

NK→ILC1-like cell plasticity was demonstrated in tumor models and *Toxoplasma gondii* infection (82–84) (**Figure 2A**). In a subcutaneous fibrosarcoma model, TGF-β signaling promoted NK→ILC1-like cell plasticity (83). Interestingly, NK cells in the tumor produced high levels of IFNγ but low levels of TNF compared to ILC1s, which correlated with antitumor activity of NK cells (83). These results are in line with other studies where the protective role of IFNγ-producing intratumoral NK cells was demonstrated whereas TNF facilitated tumor growth and metastasis (84–89). The ability of ILC1-like cells to convert back to NK cells remains to be proven experimentally.

**Figure 3:**
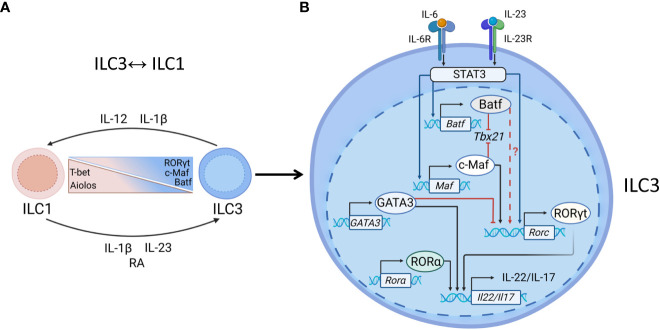
Transcriptional regulation of ILC3s. **(A)** ILC1↔ILC3 plasticity. Enteric pathogens, such as *Salmonella* and *Campylobacter* can induce ILC3→ILC1 plasticity. ILC3→ILC1 conversion is driven by IL-12 and IL-1β, produced by DCs and macrophages which leads to downregulation of RORγt and upregulation of T-bet expression. The reverse ILC1→ILC3 conversion can be induced by IL-23, IL-1β and retinoic acid (RA). Aiolos, c-Maf and Batf regulate ILC3↔ILC1 plasticity: Aiolos maintains ILC1 phenotype whereas c-Maf and Batf promote ILC3 phenotype. **(B)** Maintenance of ILC3 identity. RORγt is critical for NCR^+^ ILC3s to produce IL-22 and IL-17. IL-23 binds to IL-23R to activate STAT3, inducing RORγt. IL-6-IL6R signaling can induce STAT3 activation. Activated STAT3 induces transcription of c-Maf and/or Batf, each of them can bind to T-bet locus bind to T-bet locus, preventing T-bet transcription and acquisition of ILC1 phenotype. Batf and c-Maf individually or synergistically induce RORγt expression in T cells, however their ability to drive RORγt expression in ILC3s has not been shown yet. GATA3 limits RORγt expression by direct binding to RORγt gene locus. GATA3 and RORα can cooperate with RORγt to induce IL-22 production.

Studies in mouse models showed that conversion of ILC3s to IFNγ-producing ILC1s is controlled by downregulation of RORγt and upregulation of T-bet and is induced by IL-12, IL-15, and IL-18 (18, 19, 73, 81) (**Figure 3A**). Similarly, ILC3→ILC1 plasticity in humans is controlled by IL-1β and IL-12, whereas combination of IL-23 and IL-1β can reverse ILC1→ILC3 conversion, with retinoic acid further amplifying this reverse plasticity (18, 90). It has been previously proposed that ILC3↔ILC1 plasticity can only occur in the adult intestine (18, 67, 73); however, a recent study showed the presence of ILC1s and ILC3s with a previous history of RORγt or T-bet expression, respectively, during embryonic development (91). This plasticity is likely driven by the changing tissue microenvironment during intestinal tissue development.

Conversion of NCR^+^ to NCR^-^ ILC3s has been described in the intestine (67, 73, 92, 93). The differentiation of NCR^-^ to NCR^+^ ILC3 is driven by Notch signaling and depends on T-bet (67, 92, 94). Notch signaling in combination with microbial cues and IL-23 instructs the upregulation of T-bet, thereby regulating the development of NCR^+^ ILC3s (67, 95). Interestingly, some NCR^-^ ILC3s transiently expressed NCR (92, 93), suggesting the plasticity within ILC3 subsets. In contrast to Notch signaling, TGF-β prevents generation of NCR^+^ILC3s from their NCR^-^ ILC3s precursors (92, 93).

A delicate balance between T-bet, GATA3 and RORγt determines the fate of ILC subsets. Computational analysis of interactions between TFs and their target genes showed that LDTFs can antagonize each other thereby regulating ILC fate (7). Thus, T-bet represses LDTFs in both ILC2s and ILC3s (7). In turn, RORγt antagonizes NK and ILC1 transcription factors T-bet and Bach2 (7). The balance between T-bet, GATA3 and RORγt also defines the developmental fate of NCR^-^ILC3s, NCR^+^ ILC3s or ex-ILC3s (T-bet^+^ ILC1s with a previous history of RORγt expression) (10, 67). Since GATA3 limits RORγt expression by directly binding to *Rorc* but not *Tbx21* gene in NCR^+^ILC3s (10), it is possible that the ratio between GATA3 and RORγt expression regulates NCR^+^ILC3→ILC1 plasticity. Reduced RORγt expression allows expression of genes associated with type 1 immunity (4, 67). In turn, T-bet expression is also required for ILC3→ILC1 plasticity as ILC3s with deletion of both T-bet and RORγt failed to acquire ILC1-like phenotype (4). Interestingly, complete ILC3→ILC1 transition requires downregulation of both RORγt and RORα (4). These studies indicate that the balance between ILC3s and ILC1s is tightly regulated by a network of TFs through direct control of effector genes or through indirect regulation of other factors that can promote or restrain ILC plasticity.

**Figure 4:**
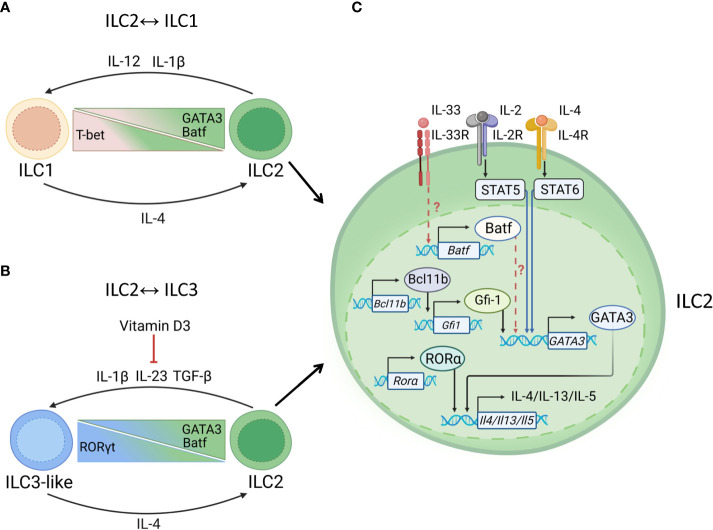
Transcriptional regulation of ILC2s. **(A)** ILC1↔ILC2 plasticity. Mucosal pathogens, such as *M. tuberculosis* can induce production of IL-12, IL-1β to drive ILC2→ILC1 plasticity. ILC2→ILC1 conversion is controlled by downregulation of GATA3 and upregulation of T-bet. Batf supports ILC2 maintenance. ILC2→ILC1 conversion can be reversed by IL-4. **(B)** ILC2↔ILC3 plasticity. Helminths, such as *N. brasiliensis* induce IL-1, IL-23, and TGF-β to promote ILC2→ILC3 conversion. Upregulation of RORγt results in acquisition of ILC3-like phenotype. Vitamin D3 prevents the conversion of ILC2 into IL-17 producing ILC3-like cells, possibly by reducing IL-23R expression on ILC3s and limiting acquisition of RORγt. ILC2→ILC3 conversion can be reversed by IL-4, which induces GATA3 expression. **(C)** Maintenance of ILC2 identity. GATA3 is critical for type 2 cytokine production: IL-5, IL-4, IL-13. IL-4 binds to IL-4R leading to activation of STAT6 which induces GATA3 transcription. GATA3 induces production of IL-5, IL-13, IL-4, IL-9. GATA3 can increase expression of IL-33R which leads to Batf upregulation. Batf may further support maintenance of ILC2 phenotype by promoting GATA3 expression. STAT5 activation by IL-2 and IL-7 can upregulate GATA3 expression. In addition to GATA3, RORα support IL-13 and IL-5 production by ILC2s. Bcl11b may support ILC2 phenotype by suppressing Ahr and promoting Gfi1, RORα and GATA3 expression.

Distinct cytokines regulate ILC2 plasticity (43, 45). Thus, stimulation of ILC2s with IL-12 and IL-1β resulted in IFNγ production, accompanied by downregulation of GATA3 and upregulation of T-bet (43, 96). This conversion can be reversed by IL-4 (**Figure 4A**). IL-1β, IL-23 and TGF-β promote production of IL-17 by ILC2s with increased expression of RORγt (45) (**Figure 4B**).

Taken together, recent studies identified the network of TFs which cooperate with core LDTFs to control ILC effector functions and their identity. Therefore, in the next sections of this review we will focus on recent advances in understanding the potential mechanisms maintaining the transcriptional identity of ILC subsets.

## 4 Transcriptional regulation of ILC1 identity

Cell surface cytokine receptors on ILCs activate broadly expressed transcription factors such as STATs which control ILC effector programs (97). For instance, activation of STAT4 *via* IL-12R signaling promotes IFNγ production by NK cells and ILC1s, while (98) which promotes IFNγ production by NK cells and ILC1s (99–101), while STAT3 activation *via* IL-23R controls IL-22 and IL-17 production by ILC3s (102) (**Figure 2B**). The potential contribution of STAT signaling to ILC1→ILC3 plasticity is highlighted by recent work demonstrating that STAT4 expression correlates with T-bet expression in ILC1s in the small intestine (103) (**Figure 2B**). Furthermore, distinct ILC subsets have different basal levels of STAT4 with the highest expression in ILC1s and NK cells, whereas CD4^+^ ILC3s display low level of STAT4 expression (103). Interestingly, ILC1s and NCR^+^ ILC3s have similar expression level of STAT4 (103). Furthermore, IL-23 and IL-12 can differentially activate STAT3 and STAT4 in ILC1s and NCR^+^ ILC3s. Thus, IL-23 signaling activates both STAT4 and STAT3 in NCR^+^ ILC3s but not in ILC1s, where activation of STAT4 and STAT3 is induced by IL-12 (103). In contrast, another study showed that IL-12 activates STAT4 in both NCR^+^ ILC3s and ILC1s, isolated from the colon (104). These differences could be due to the different responsiveness of ILCs from the small intestine and the colon to IL-12. Since IL-12 and IL-23 receptors share p40 subunit (105), it is possible to hypothesize that both cytokines could activate STAT4 signaling, driving IFNγ production by ILC1s and NCR^+^ ILC3s. In line with this, activated STAT4 binds to *ifng* promoter and activates its transcription (106, 107). Importantly, IL-23 stimulation induces chromatin accessibility of *ifng* gene in NCR^+^ ILC3s (103) indicating that the ability of NCR^+^ ILC3s to produce IFNγ in response to IL-23 partially depends on STAT4 activation. T-bet can cooperate with Runx3 to promote IFNγ production in T cells (108). Additionally, another study showed that Runx3 can induce IFNγ production in ILC1s and ILC3s, potentially *via* formation of transcriptional complex between Runx3 and T-bet (109)(**Figure 2B**). Therefore, it is possible that cooperation of T-bet with Runx3 and STAT4 promotes ILC1→ILC3 plasticity by increasing remodeling and accessibility of *ifng* locus and making cells more responsive to IL-12. (**Figure 2B**). Further studies are needed to define whether IL-12 dependent STAT4 activation leads to ILC1 plasticity.

## 5 Transcriptional regulation of ILC3 identity

RORγt is critical for the maintenance of ILC3 phenotype (**Figures 3A, B**) (64, 110, 111). IL-23/IL-6-mediated STAT3 activation induces IL-17 and RORγt expression (112–114). STAT3 can also directly bind to IL-22 locus to induce its transcription in response to mucosal pathogen *Citrobacter rodentium* (*C. rodentium*) *(* 102). Furthermore, RORγt and STAT3 can further cooperate with Ahr to amplify IL-22 production (102, 115).

Transcription factor c-Maf is a known regulator of *Rorc* in T cells (114). Recently, transcriptomic analysis of ILCs revealed the expression of c-Maf along with RORγt in NCR^+^ ILC3 (11, 116, 117) whereas CCR6^+^ ILC3s had the lowest c-Maf expression (7, 116). Interestingly, c-Maf expression correlates with T-bet, suggesting the potential role of c-Maf in regulation of NCR^+^ ILCs (116). Importantly, loss of c-Maf led to higher numbers of NCR^+^ ILC3s with the increased IFNγ but decreased IL-22 production in the intestine (7, 116) implying that c-Maf regulates T-bet expression to maintain the ILC3 phenotype (**Figure 3B**). Indeed, ATAC-seq analysis revealed that c-Maf prevents NCR+ ILC3s to acquire ILC1-phenotype by direct binding to *Tbx21* promoter to attenuate the expression of T-bet (116, 117). Consistent with the induction of RORγt in T cells, c-Maf can directly activate RORγt transcription in NCR^+^ ILC3s (114, 116, 117) (**Figure 3B**). Recently it has been shown that IL-1β and IL-18 induce c-Maf expression through NF-κB signaling (116, 117), as pharmacological inhibition of NF-κB abrogated the cytokine-induced c-Maf expression in NCR^+^ ILC3s (117). However, how NF-κB signaling regulates c-Maf expression remains to be further investigated. Notch signaling can also regulate c-Maf expression, as c-Maf expression in ILC3s is higher in the presence of Notch ligand (116, 117).

IL-22 production by ILC3 is regulated by RORγt in cooperation with other TFs (4, 111, 115). A recent study demonstrated that interferon regulatory factor 1 (IRF-1) controls IL-23 induced production of IL-22 by ILC3s during *C. rodentium* infection (118). Another example of TF that regulates function of ILC3s during inflammation is ZBTB46 (119) (**Figure 3B**). Although ZBTB46 was previously described as a critical TF for the classical dendritic cells development (120, 121), a recent study revealed that ZBTB46 is also expressed by CCR6^+^ ILC3s (119). The ZBTB46-expressing ILC3s are a primary source of IL-22 in the large intestine and are required for protection against *C. rodentium* (119). Inactivation of ZBTB46 in ILC3s promoted *C. rodentium*-induced intestinal inflammation and bacterial load, but did not affect IL-22 expression (119). Instead, CCR6^+^ ILC3s in these mice exhibited a proinflammatory phenotype with increased expression of OX40L (*Tnfsf4*) and PTGS2 (119). Interestingly, *Zbtb46* locus has the RORγt binding sites suggesting that RORγt regulates ZBTB46 expression in CCR6^+^ ILC3s (119). Additionally, microbiota controls ZBTB46 expression in ILC3s as germ-free mice exhibited high ZBTB46 expression which was downregulated after colonization with conventional microbiota (119). Thus, these data suggest that RORγt regulates ZBTB46 expression, which is required to restrain proinflammatory functions of ILC3s. However, the connection between ZBTB46 and RORγt in other models of intestinal inflammation remains to be determined. As ZBTB46 is overexpressed in inflamed tissue of IBD patients (119), ZBTB46 targeting strategies could have a therapeutic potential to inhibit intestinal inflammation.

## 6 Role of RORα in ILC3 identity

Although early studies showed the critical role of RORα in ILC2 development (55, 122), recent studies suggest that RORα cooperates with RORγt for the maintenance of ILC3 phenotype and effector functions (4, 49, 123) (**Figure 3B**). Thus, it has been demonstrated that in the absence of RORγt expression, ILC3s are still capable to produce IL-17 and IL-22 in the small intestine, suggesting that other TFs can cooperate with RORγt to regulate ILC3 effector functions (4, 124). RORγt and RORα differentially regulate the production of IL-17 and IL-22 in distinct ILC3 subsets (4). Deletion of both RORα and RORγt in mice using inducible Id2-Cre resulted in the loss of most ILC3 markers, but preserved IL-22 production by LTi-like cells and CCR6^-^NCR^-^ ILC3s, but not by NCR^+^ ILC3s (4). Conversely, IL-17 production was reduced in all ILC3 subsets in the absence of either one or both TFs (4). Furthermore, RORγt cooperates with RORα to prevent NCR^+^ ILC3s from acquiring an ILC1-like phenotype, as the deletion of both TFs leads to transition of NCR^+^ ILC3s to ILC1s in T-bet dependent manner. However, simultaneous deletion of T-bet and RORγt prevented NCR^+^ ILC3→ILC1 transition (4). RORα can sustain IL-22 production by ILC3s through the maintenance of IL-23R expression, which, together with IL-1R, contributes to IL-22 production in ILC3s (73, 110, 125). Consistent with this, simultaneous deletion of RORα, RORγt and T-bet caused the complete loss of IL-22 production in all ILC subsets (4). These data indicate that RORα together with RORγt maintains the ILC3 effector program in NCR^+^ ILC3s, whereas T-bet supports type 1 effector program. Thus, the role of these TFs in ILC development is distinct from their role in maintaining ILC effector functions.

## 7 Role of Batf in ILC3 identity

Batf is another TF which controls development of effector T cells, as well as ILCs (5, 126). Batf-binding motifs can be found in regulatory regions of the genes critical for T cell development, such as T-bet and Eomes (127). During Th17 cells development, Batf cooperates with IRF4 to promote chromatin accessibility and recruitment of RORγt (128). Although all mature ILCs express Batf (126), analysis of chromatin landscape in different ILC subsets revealed the enrichment of Batf motif only in ILC2 and ILC3 subsets (5), indicating a potential role of Batf in regulation of ILC3 and ILC2 functions. Transcription factor Batf was shown to promote ILC3 phenotype since Batf deficiency resulted in increased numbers of IFNγ-producing ILC3s in the small and large intestine (129). Similar to c-Maf, Batf maintains ILC3 phenotype by binding to *Tbx21* locus and preventing formation of Runx3 and T-bet complex thereby preventing acquisition of ILC1 phenotype (116, 129). Additionally, Batf inhibits chromatin accessibility of IL-1R, IL-12Rβ and IL-18RAP genes, leading to the reduced responsiveness to ILC1 phenotype-driving cytokines (129). In contrast to (129), another study showed that Batf ablation led to the reduction of ILC1 and ILC2 subsets in the colon with unaltered numbers of T-bet^+^ ILC3s in naïve mice (126). Moreover, IFNγ production by ILC1s after *C. rodentium* infection was reduced in the small intestine but not in the colon of Batf-deficient mice compared to controls (126). Furthermore, Batf deficiency led to reduced IL-22 production accompanied by reduced numbers of RORγt^+^ ILC3s (126). These results suggest that Batf regulates ILC functions in the gut during homeostatic conditions and in infectious disease. It seems that the role of Batf in regulation of ILC3 homeostasis is broader than the role of c-Maf, as Batf not only restricts ILC3→ILC1 plasticity but also restrains CD4^+^ T cell activation by inducing MHCII expression on ILC3s in the small intestine (129). The different outcomes of Batf ablation in these studies could be due to the distinct regulatory mechanisms in physiologically different parts of the small and large intestines. For example, microbiota composition is less diverse and abundant in the small intestine compared to the colon which could potentially change Batf-dependent regulation of ILCs (130). Additionally, it is known that Batf regulates microbiota in the small intestine by shifting the proportion of beneficial and potentially pathogenic microbiota species that may in turn change ILCs composition (129). Batf regulates differentiation of Th17 cells and promotes IL-17 expression by binding to the promoter of *Il17* gene (131, 132). As IL-6/STAT3 signaling is known to induce Batf-dependent expression of c-Maf in follicular helper T cells (133, 134), it is tempting to speculate that Batf can cooperate with c-Maf in NCR^+^ ILC3s to promote ILC3 phenotype by maintaining sustainable expression of RORγt (**Figure 3B**). Further studies are needed to define the mechanism by which Batf and c-Maf regulate the balance of ILC3s and ILC1s in the gut.

## 8 Role of Ikaros TF members in ILC3 identity

Several studies identified the potential role of Ikaros family of transcription factors in ILC maintenance (90, 135). Analysis of expression of Ikaros family members in ILCs revealed the highest levels of Helios in ILC3s (90, 135), whereas Ikaros is expressed in all ILC populations (135), with the lowest expression in intestinal ILC3s (136). Ikaros suppresses effector functions of mature ILC3s in the gut by interacting with Ahr and inhibiting its translational activity (136). Another member of Ikaros family, Aiolos, is expressed in all ILCs in mice, except LTi-like cells (4). Aiolos regulates differentiation to type 1 program, as high levels of Aiolos were detected in ILC1s and NK cells but not in ILC3s and ILC2s (135). Furthermore, in the presence of lenalidomide, an immunomodulatory drug which selectively degrades Ikaros and Aiolos (137, 138), reduced IL-12 mediated ILC3→ILC1 plasticity and increased IL-22 expression were reported (90). Thus, these results suggest a potential role of Aiolos in promoting ILC3→ILC1 plasticity (90). Consistent with this, microarray analysis showed that lenalidomide treatment upregulated ILC3-related genes in tonsillar ILC3s treated with IL-12 and IL-1β (90), suggesting that Aiolos and Ikaros may regulate the balance between ILC1s and ILC3s *via* suppression of ILC3-related genes and promotion of ILC1-specific genes.

## 9 Transcriptional regulation of ILC2 identity

Recent evidence from studies in mice and humans reported that ILC2s can also adopt alternative fates and convert to IFNγ producing ILC1s (43, 139–141) (**Figure 4A**). Specifically, stimulation of isolated ILC2s *in vitro* with IL-1β+IL-12 or IL-33+TSLP+ IL-12 resulted in robust IFNγ production (43) whereas IL-1β or IL-33 alone induced IL-5, IL-4, GM-CSF but not IFNγ (43, 141). However, activation of ILC2s in the presence of IL-1β induced low levels of T-bet and IL-12R promoting ILC2→ILC1 plasticity in response to IL-12 mediated STAT4 activation (141). Moreover, IL-12 stimulation led to reduction of GATA3 and subsequent induction of T-bet expression in ILC2s (43, 141). These results demonstrate that ILC2s can transdifferentiate into ILC1s in response to type 1 inflammatory cytokines. It has been shown that ILC2→ILC1 plasticity can be reversed by IL-4 (43, 45, 142), suggesting that the ratio between IL-12 and IL-4 regulates the functional identity of ILCs. Interestingly, IL-4 stimulation led to inhibition of ILC2→ILC3 transition along with inhibition of STAT3 activation indicating a potential role of STAT3 in promoting ILC2 plasticity (142). Consistently, another study showed that ILC2s can convert to RORγt-expressing, IL-17 producing ILC3s in the presence of IL-1β, IL-23 and TGF-β (45). In addition to IL-4, vitamin D3 can prevent ILC2→ILC3 plasticity (142). Since vitamin D3 downregulates the expression of IL-23R on ILC3s and consequently IL-23-dependent production of IL-22 and IL-17 (143), it is possible that vitamin D3 limits the acquisition of RORγt.

GATA3 supports ILC2 phenotype by inducing type 2 effector cytokines: IL-5, IL-4, IL-13 along with IL-33 receptor (also known as ST2) and by suppressing alternative cell lineage genes (53, 144, 145). Given that GATA3 directly binds to the *Il4/Il13* locus utilizing similar mechanism described for Th2 cells, it is possible that IL-4 reverses ILC2 plasticity through STAT6-induced GATA3 expression (54, 146, 147). Moreover, IL-2, IL-7, and TSLP can synergistically induce GATA3 through STAT5 activation (145, 148, 149) (**Figure 4B**). It was shown that STAT5 binds to GATA3 promoter and directly upregulates its expression (145). It should be noted that GATA3 can autoregulate its own transcription by binding to GATA3 gene locus in T cells (54, 150). Accordingly, similar mechanism of GATA3 regulation was proposed for ILC2s (151).

Current studies in mouse models indicate the existence of ILC2 subsets that differently respond to IL-25 and IL-33 in the lungs (40, 152, 153). Natural ILC2s (nILC2) are IL-33 responsive and can be found in the lungs in homeostatic conditions, whereas inflammatory ILC2s (iILC2) respond to IL-25 and emerge after IL-25 treatment or during helminth infection (40, 152, 153). iILC2s migrate from the small intestine to the lungs during early stages of *Nippostrongylus brasiliensis* (*Nb*) infection, where they initiate type 2 immune response by producing IL-13 (152). At the later stages of helminth infection, iILC2s can become responsive to IL-33 and give rise to nILC2s (40). Additionally, iILC2s express RORγt along with GATA3 and produce IL-17 together with type 2 cytokines during helminth and *Candida albicans* infections (40, 152, 153). The iILC2 phenotype is regulated by Notch signaling (153). Notch *via* formation of complexes with transcription factors directly promotes RORγt expression in iILC2s without affecting GATA3 expression (153). Interestingly, Notch signaling in the presence of IL-33 inhibits the proliferation and activation of nILC2s thereby promoting iILC2s in the lungs during inflammation (153). In contrast to iILC2s, nILC2s are tissue-resident cells that produce more IL-9 after activation (152). IL-33 and TSLP activate IL-9 production by ILC2s which further induces IL-5 and IL-13 during helminth infection (41, 42, 154). Since ILC2s express IL-9R, ILC2-derived IL-5/IL-13 can be regulated in autocrine manner by IL-9 (41, 154). Similar to T cells, IL-9 production in ILC2s depends on the transcription factor IRF4 (42, 155). Accordingly, IRF4-dependent production of IL-9 in ILC2s facilitates rapid initiation of the immune response against helminth pathogens.

The role of Batf in the maintenance of ILC2 phenotype has also been described (126, 156). Ablation of Batf led to defective production of IL-5 and IL-13 by ILC2s in the lungs suggesting that Batf expression is required for proper ILC2 activation (126, 156). Additionally, during *Nb* infection Batf regulates expression of IL-4 and IL-13 in iILC2s but not in nILC2s (157). Upon viral infection, Batf-deficient ILC2s upregulate genes associated with ILC3 phenotype, such as *Il23r*, *Il17a*, *Il6ra* and *Il1b* (156). Since Batf deficiency reduced GATA3 and RORα expression in ILC2s (156), it remains to be determined whether Batf controls expression of ILC2 lineage-defining TFs directly or through the formation of transcriptional complexes with other factors.

Similar to Batf, Bcl11b and Gfi-1 are the transcription factors maintaining ILC2 identity by promoting type 2 effector program and repressing ILC3 associated genes (158) (**Figure 4C**). The ablation of either Bcl11b or Gfi-1 led to reduced GATA3 and increased RORγt expression (158, 159). Additionally, Bcl11b deficiency resulted in downregulation of the genes controlling ILC2s effector functions, such as *RORα, Gfi-1* and *ST2*, and concomitant upregulation of *Ahr* and *Il23r* (158). Ahr has been shown to suppress ILC2 effector functions in the gut by downregulating Gfi-1-ST2 pathway (160). Given that Ahr is important for ILC3 maintenance and function (67, 102, 115), Bcl11b can suppress ILC3s by directly binding to Ahr promoter (158, 161, 162). Additionally, Bcl11b promotes ILC2 identity by inducing Gfi-1 expression which stabilizes GATA3 expression (158, 159). At the same time, Bcl11b suppresses ILC3 effector program in ILC2s by repressing Ahr expression (158).

IL-33 pathway serves as an additional regulator of the ILC2 identity (**Figure 4C**). IL-33 signaling is an important activator of ILC2s and Th2s (48, 163). It has been shown that Bcl11b, Gfi-1 and Batf directly bind to the promoter of the *Ilrl1* gene to support IL-33R expression (156, 158, 159). These data suggest that the positive regulation of IL-33R expression by Bcl11b, Gfi-1 and Batf can promote ILC2 phenotype stability. It remains to be determined whether these transcriptional networks control ILC2→ILC3 plasticity during inflammation. Since the regulation of ILC2 phenotype in the intestine is different from the lungs (57), further studies are needed to elucidate the molecular mechanisms of TF regulation in ILCs in different tissues under physiological and pathological conditions. Recently developed mouse model in which ILC2-specific NMUR1 promoter drives the expression of Cre recombinase allows to selectively target ILC2s (164, 165). These mice will be helpful to study the specific role of different TFs in ILC2 maintenance and plasticity.

## 10 Conclusions and perspectives

Recent studies have led to great progress in the characterization of ILC development and plasticity. An important feature of ILCs is their ability to quickly respond to the changing environment caused by tissue damage, pathogen invasion or cell stress. The prompt ILC response is mediated by rapid cytokine production to promote protection against harmful stimuli. Emerging studies report that most ILC subtypes exhibit plasticity and can acquire phenotype of another ILC subset in response to environmental changes. ILC plasticity could be one of the mechanisms enabling rapid response to pathogenic stimuli. Transcriptome analysis of ILCs revealed the existence of intermediate transcriptional profiles within every main ILC subset.

ILC identity is controlled by transcription factors. Transcriptome studies revealed the similarities between mechanisms regulating effector programs in T cells and ILCs. Like T cells, ILCs depend on the same lineage-determining transcription factors T-bet, Eomes, GATA3, RORα, RORγt for their development and function. It is becoming increasingly clear that complex transcriptional networks that regulate ILC identity utilize similar gene-regulatory mechanisms compared to T cells. It is well established in the literature that core transcription factors are required to control ILC effector functions, but recent studies also highlighted that the ratio between different transcription factors determines ILC identity and phenotype maintenance. Moreover, the role of TFs also depends on the developmental stage of ILCs. Some transcription factors are critical to determine ILC fate, while other TFs define phenotype of mature ILCs. Emerging data suggests that tissue microenvironment can also impact the expression of genes which define ILC phenotypes. Epigenetic studies uncovered the differences in regulomes between ILCs and T cells during development. However, more work is required to fully understand how different transcription factors and epigenomic elements control gene expression and lineage specificity in response to different pathogenic stimuli. Mechanisms of epigenetic control of ILC plasticity are still poorly characterized. Revealing how chromatin landscape is changed in ILCs in response to different stimuli may uncover previously unrecognized mechanisms of transcriptional control of ILC functions as well as their phenotype.

Although ILCs are present in human tissues and contribute to the host protection, it is still poorly understood how ILC identity is regulated in humans. The changes in ILC composition have been described in human diseases. However, it remains to be determined whether changes in ILC composition occur because of ILC plasticity or mediated by mature or immature precursors during inflammation. The regulatory mechanisms and the functional role of ILC plasticity during ontogenesis remains to be explored. Finally, understanding the regulation of transcription factor networks in ILCs could uncover new therapeutic targets to treat autoimmune diseases and chronic infections.

## Author contributions

Study concept and design: AK, AT. Wrote and edited manuscript: AK, SS, EK, AT. All authors contributed to the article and approved the submitted version.
